# The development and testing of the quality use of medications in dementia (QUM-D): a tool for quality prescribing for behavioral and psychological symptoms of dementia (BPSD)

**DOI:** 10.1017/S1041610214002816

**Published:** 2015-02-02

**Authors:** Carmelle Peisah, Julie-Anne Strukovski, Chanaka Wijeratne, Rosalind Mulholland, Georgina Luscombe, Henry Brodaty

**Affiliations:** 1 Specialist Mental Health Services for Older People, Mental Health Drug and Alcohol, Northern Sydney Local Health District, Sydney, Australia; 2 University of Sydney, Sydney, Australia; 3 University of NSW, Sydney, Australia; 4 Dementia Collaborative Research Centre, University of New South Wales, Sydney, Australia

**Keywords:** BPSD, prescribing, quality, dementia, medications

## Abstract

**Background:**

Behavioral and psychological symptoms of dementia (BPSD) are virtually ubiquitous in dementia. Excessive recourse to use of psychotropics which have high risk to benefit ratio remains a global problem. We aimed to identify components of quality prescribing in BPSD to develop a tool for quality prescribing and to test this tool.

**Methods:**

We used Delphi methodology to identify elements of quality prescribing in BPSD. The tool was tested by a range of medical and nursing professionals on 48 patients, in inpatient and ambulatory settings in Northern Sydney Local Health District, Australia.

**Results:**

Consensual opinion using Delphi method was that quality prescribing in dementia comprised ten factors including failure to use first line non-pharmacological strategies, indication, choice of drug, consent, dosage, mode of administration, titration, polypharmacy, toxicity, and review. These elements formed the quality use of medications in dementia (QUM-D) tool, lower scores of which reflected quality prescribing, with a possible range of scores from 0 to 30. When inter-rater reliability was tested on a subgroup of raters, QUM-D showed high inter-rater reliability. A significant reduction in QUM-D scores was demonstrated from baseline to follow-up, mean difference being 5.3 (SD = 3.8; 95% confidence interval 4.1–6.4; *t* = 9.5; df = 47; *p* < 0.001). There was also a significant reduction in score from baseline to follow-up when rated by clinical nurse consultants from a specialized behavior assessment management service (BAMS) (*N* = 12).

**Conclusion:**

The QUM-D is a tool which may help to improve quality prescribing practices in the context of BPSD. In this setting, we consider quality prescribing, and accordingly the obligations of prescribers, to be an inclusive concept rather than just adding to the mantra of “not prescribing.”

## Introduction

BPSD (Burns *et al.*, 2012) occur so commonly as to be virtually ubiquitous in this disease (Brodaty *et al.*, 2001). Reliance on medications to “manage” these symptoms occurs commonly and is increasing, at least in Australia. In Sydney nursing homes 47.5% of residents were being prescribed psychotropic medications in 2009, an increase of 19% compared with an earlier audit in 2003 (Snowdon *et al.*, 2011). This rate varies (Hyojeong and Whall, 2006; Rochon *et al.*, 2007) depending on whether all nursing home residents are sampled or only those with dementia, of whom 75% were prescribed psychotropics in one study (Selbaek *et al.*, 2007).

Based on limited efficacy (an effect size of 0.13–0.20 for antipsychotics)(Schneider *et al.*, 2006; Ballard *et al.*, 2009a), discontinuation studies (Ballard *et al.*, 2008; Ballard and Corbett, 2010; Devanand *et al.*, 2012; Declercq *et al.*, 2013) and a growing body of evidence for non-pharmacological interventions (Chenoweth *et al.*, 2009; O’Connor *et al.*, 2009; Brodaty and Arasaratnam, 2012; Chenoweth *et al.*, 2014; Testad *et al.*, 2014), it has been estimated that two thirds of these prescriptions were unnecessary (Ballard *et al.*, 2014). Moreover, the significant side effect burden (Byerly *et al.*, 2001; Hedges, 2003; Katz *et al.*, 2004; Hien *et al.*, 2005; Schneider *et al.*, 2006; Ballard, 2009b; Banerjee *et al.*, 2011), including an association with increased mortality (Brodaty *et al.*, 2003; Schneider *et al.*, 2006; Ballard *et al.*, 2011; Kales *et al.*, 2012; Huybrechts *et al.*, 2012; Gerhard *et al.*, 2014) mandates a continued push to improve the quality and safety of prescribing. In this context, the problem of prescribing extends beyond antipsychotics to the use of psychotropics in general (Burns *et al.*, 2012), particularly in the light of recent reviews and trials suggesting lack of efficacy of valproate for BPSD (Lonergan and Luxenberg, 2009) and antidepressants for depression in dementia (Weintraub *et al.*, 2010; Banerjee *et al.*, 2011).

This increase in Australian prescribing data contrasts sharply with the reduction reported elsewhere, such as in the United Kingdom (Health and Social Care Information Centre, 2012) where, in 2009, a government-driven imperative to reduce unnecessary prescribing was triggered by a report for the Department of Health (Banerjee, 2009). Initiatives such as this, together with the French Survey and the National BPSD Swedish Registry, have been part of ALCOVE (Alzheimer Cooperative Evaluation in Europe; http://www.alcove-project.eu), a key collaborative benchmarking project involving European member states with 16 associated partners which has developed a number of concrete tools and supports to tackle inappropriate prescribing.

Several intensive studies of the effects of multidisciplinary teams on prescribing in nursing homes are underway. For example, in the Netherlands, the PROPER II study (PRescription Optimization of Psychotropic drugs in Elderly nuRsing home patients with dementia) is being conducted by pharmacists, physicians, and nurses and has three components: preparation and education, conduct, and evaluation/guidance. Primary outcome measures include percentages of patients with appropriate psychotropic drug use and secondary outcomes include overall frequency of psychotropic drug use, neuropsychiatric symptoms, quality of life, activities of daily living, side effects, and mortality (Smeets *et al.*, 2013). In Australia, the HALT (halting antipsychotic use in long term care) (Brodaty *et al.*, 2014) and the RedUSe studies (http://www.utas.edu.au/pharmacy/pharmacy-news/news/utas-to-take-innovative-sedative-reduction-program-national) are using similar methodologies of multidisciplinary support teams of nurses, psychiatrists, general practitioners, and pharmacists to reduce the inappropriate use of antipsychotic medication in residential care facilities.

However, as noted recently by Ballard *et al.* (2014, p4):

“despite the reductions in antipsychotic use, there are still a significant number of people with dementia prescribed antipsychotics and it is important that we continue to use research evidence to improve the safety and quality of prescribing.”

While there are tools which measure inappropriate drug choice, polypharmacy or drug burden in older people (Beers, 1997; Hillmer *et al.*, 2007; Pham and Dickman, 2007; Fick *et al.*, 2008; Haque, 2009; Hamilton *et al.*, 2011), these are lists of potentially harmful drugs or burden indexes with validation, rather than efficacy data in terms of physician prescribing behavior. More importantly, there are no tools that address the broader concept of quality prescribing in the context of dementia, and specifically, in the unique setting of BPSD.

We aimed to: (i) identify, using expert opinion, the components of quality prescribing in dementia and BPSD; (ii) use this expert opinion to develop a tool for education and quality prescribing in dementia; (iii) test this tool; and in particular, (iv) test its efficacy when used by clinical nurse consultants from a specialized Behavior Assessment Management Service (BAMS).

## Methods

### Tool development

We used the Delphi method to identify the elements of quality prescribing in dementia and BPSD. The Delphi method is a flexible, effective, and efficient research method to collect and distill expert opinion using a series of consultations and feedback, particularly suited to improving understanding of problems or concepts (Skulmoski *et al.*, 2007). We used an iterative (i.e. evolving) multi-stage group facilitation process, designed to transform opinion into group consensus (defined here as at least 90% concordance) achieved through a series of five Delphi rounds where information was fed back to participants or “panel members.” Panel members included five old age psychiatrists, three pharmacists, one pharmacologist, and three geriatricians from across Sydney with extensive knowledge and experience in the area of dementia, BPSD, and medications. The round one question was “What is quality prescribing in dementia, and what is not?,” responses to which were shared in subsequent rounds, feedback collated, and modified to achieve an inclusive and collective perspective of the factors which comprised the final QUM-D tool. The focus of the subsequent rounds, where some of the factors were expanded to include sub-factors, was to transform each of these factors (e.g. “Polypharmacy,” “Failure to obtain consent”) into consensually derived, operationalized descriptors (e.g. > 2 psychotropics being used simultaneously was considered outside best practice). Participants’ responses were verified and opinions were fed back and directed to the other participants, each of whom were given an opportunity to comment on the items and the emerging consensual final tool.

### Tool testing

The tool (Appendices A and B) was tested by a range of medical and nursing professionals on 48 patients, in a range of inpatient and ambulatory settings (see Table 1) in the Northern Sydney Local Health District (NS-LHD), Australia. The tool was specifically tested in the BAMS of the NS-LHD by clinical nurse consultants. The remit of the services upon which the tool was tested was to treat patients with severe to extreme BPSD, such as dangerous physical aggression, according to an Australian hierarchical model of service delivery which rates BPSD severity and service need (Brodaty *et al.*, 2001).

**Table 1.** Characteristics of raters and settings for the testing of QUM-D tool

	***N* =** 48
Setting	Nursing home *N* = 37
	Ambulatory community at home *N* = 7
	Inpatient = 4
Rater	Old age psychiatrist = 31
	Registrar in training = 4
	BAMS^ab^ clinician *N* = 12
	Geriatrician = 1

Key ^a^Behavior Assessment Management Service clinical nurse consultants.

^b^In liaison with general practitioner.

Each rater was asked to score the QUM-D upon initial assessment of any patient with dementia referred for treatment of BPSD who are currently prescribed psychotropics and then subsequently after intervention, prior to discharge from care, with the requested aim of reducing the score (see Appendix B). Because the focus was on prescribing behavior, no demographic or clinical details about the patients were obtained for the study. Written feedback was also sought from raters from each of the disciplines regarding their use of the tool and resistances encountered to score reduction.

Scores from seven pairs of raters (a second, independent rater separate from the study-raters and blind to their results were matched for seven of the BAMS study raters) were sought to test inter-rater reliability. The second rater completed their rating based on the same information available to the first rater.

Differences between baseline and prior to discharge scores on the QUM-D were examined using paired samples *t*-tests. Inter-rater reliability was analyzed using an intraclass correlation coefficient. Analyses were conducted using SPSS v 21 and *α* was set at 0.05 for both analyses. Given the aim of developing a clinical prescribing and education tool, not a psychometric instrument measuring a single, unidimensional latent construct, the measurement of Cronbach's *α* was not appropriate (Sijtsma, 2009).

The study was approved by NS-LHD Human Research Ethics Committee.

## Results

### Delphi consensus and tool development

Consensual opinion reached using the Delphi rounds was that quality prescribing in dementia comprised ten primary factors, which we described in reverse because of the prevalence of problematic prescribing, viz:

1. “alternatives” i.e. failure to use non-pharmacological strategies as first line treatment;
2. “indication” i.e. use of drugs for inappropriate target symptoms that are unlikely to respond to psychotropics, such as screaming and wandering;
3. “choice of drug” with two sub-factors (a) use of drugs without evidence for efficacy in this setting; and (b) use of drugs otherwise contraindicated (e.g. typical antipsychotics in patients with Dementia with Lewy bodies);
4. “consent” (or lack thereof);
5. “dosage” with two sub-factors to show escalating “burden:” (a) dosages in excess of best practice; and (b) doses far in excess of best practice;
6. “mode of administration” e.g. use of depot antipsychotics to treat BPSD;
7. “titration” being (a) rapid; or (b) unreviewed;
8. “polypharmacy,” including (a) class duplication (e.g. two antipsychotic drugs); and with compounding “burden” (i.e. the more drugs the more “burden”) with (b) 2–4 psychotropics and (c) >4 psychotropics
9. “toxicity” (i.e. side effects); and
10. “review” (i.e. lack thereof).

With the ten primary factors, and five sub-factors, the tool comprised 15 items each with a categorical score of either zero or two, resulting in a potential range of poor quality prescribing from 0 to 30, where zero was the optimal score towards optimal prescribing (see Appendices A and B, for the final QUM-D tool tested in the field).

In one of the final rounds of feedback, we had difficulty reaching consensual agreement (i.e. for this issue, only 75% agreement of the group) regarding acceptable ceiling doses for antipsychotics. This was driven by the emerging literature regarding the relationship between antipsychotic dose and mortality (Gerhard *et al.*, 2014) as well as the different settings of panel members for treatment of BPSD and different patient target groups, such as inpatient settings with frail elderly with high medical illness burden compared with relatively more robust residents in nursing homes. This led to the “frail elderly specifier,” which allowed for a more flexible approach to ceiling doses (i.e. a lower threshold) while still mandating the need for care in regards to capping doses (see Appendix A).

### Testing of the tool

Characteristics of raters and settings for the testing are outlined in Table 1.

### Inter-rater reliability and efficacy of QUM-D

When inter-rater reliability was tested, the two raters’ QUM-D scores had high inter-rater reliability (intraclass correlation coefficient = 1.0, 95% confidence interval 0.9–1.00; *p* < 0.001).

A significant reduction in total QUM-D scores was demonstrated when baseline QUM-D scores (mean 10.0; SD = 4.9; range 2–24) were compared with follow-up scores (mean 4.7; SD = 3.4; range 0–16). The mean difference between baseline and follow-up was 5.3 (SD = 3.8; 95% confidence interval 4.1–6.4; *t* = 9.5; df = 47; *p* < 0.001). Missing data and small cells precluded examination of performance of individual items.

For the BAMS clinical nurse consultants (*N* = 12), there was a significant reduction in score from baseline (mean 12.8; SD = 5.5) to follow-up (mean 6.0; SD = 2.8); the mean difference between baseline and follow-up was 6.8 (SD = 4.6; 95% confidence interval 3.9–9.8; *t* = 5.1; df = 11; *p* < 0.001).

### User-friendliness, feasibility, and feedback

No training was required to use the QUM-D. The QUM-D took between 4–12 minutes to complete, depending on the accessibility of prescribing information and medication charts.

Feedback from (*N* = 8) raters regarding use of the tool (the other raters did not provide feedback) suggested that resource limitation, either in the acute care setting or, in the nursing home restricted the willingness of prescribers to reduce medication. Another significant barrier was the occasional resistance of family members to medication change due to fear of the person becoming unmanageable and losing their tenure in the nursing home. Some facility staff perceived psychotropics as “dementia medication” and were thus resistant to their withdrawal or reduction. Conversely, feedback from nursing staff who used the tool suggested that it empowered their concerns about polypharmacy when working with general practitioners.

We also provide a case example to illustrate how the QUM-D was used as an interactive decision-making tool:

Mr. X is a 78 year old man with severe vascular dementia and BPSD (repeatedly stripping and aggression) in a nursing home. He was assessed by the BAMS clinical nurse consultant who rated his QUM-D as 24, including items positive for failure to gain proxy consent or use non-pharmacological strategies prior to prescribing, polypharmacy (haloperidol, risperidone, diazepam, sodium valproate) as well as doses far in excess (5mg haloperidol prn (as needed) intramuscularly up to 10mg per day), rapid titration and failure to review. The BAMS clinician contacted the local doctor, sent him the QUM-D and discussed the score with the doctor who agreed (reluctantly) to reduce the medications while the clinician implemented a range of education and person-centered interventions in the facility. The family, who were unaware of the medication use or their side effects, were contacted to discuss the medications. The QUM-D score when repeated after three months had decreased to six. The BAMS clinician is still working with the doctor and the facility to reduce the psychotropics further.

## Discussion

We have described the development of a tool to improve quality prescribing in the setting of dementia and BPSD. In doing so, we have conceptualized quality prescribing in this context as an inclusive, heterogeneous construct, incorporating a diverse range of quality indicators, beyond mere medication choice which is often used to audit prescribing in the elderly (Beers, 1997; Fick *et al.*, 2003; 2008; American Geriatrics Society, 2012). The need to expand the concept of quality/poor quality prescribing beyond inappropriate medication choice (as defined by schema such as the BEERS criteria; Fick *et al.*, 2003) has previously been recognized and driven the development of new tools such as the screening tool for older person's potentially inappropriate prescriptions (STOPP), which addressed more commonly prescribed inappropriate medications and added additional criteria such as adverse drug-drug interactions and duplicate drug class prescription (commonly encountered in the treatment of BPSD, where prescribers resort to two antipsychotic drugs; Hamilton *et al.*, 2011).

More specific to this setting of BPSD, other prescribing schema have distinguished between “valid” and “invalid” indications for antipsychotic use in nursing facility residents such as agitated behaviors that are not dangerous, or wandering (Pham and Dickman, 2007). Additionally, the mounting and compelling evidence that relates dosage to mortality rates (Rossom *et al.*, 2010; Kales *et al.*, 2012; Gerhard *et al.*, 2014) justifies the inclusion of ceiling caps in our tool, notwithstanding the difficulty in reaching consensus regarding these. Our tool also goes beyond the idea of “reducing antipsychotic use” as the goal. Our choice to broaden the focus of our work to psychotropics in general, including valproic acid, is justified by its equivalent mortality risk with risperidone (Kales *et al.*, 2012) and its lack of efficacy data (Lonergan and Luxenberg, 2009).

Quality prescribing is about more than just reducing medications. In addition to the risk and implications of adverse drug events, in the setting of BPSD there is the additional problem of unnecessary prescribing. Specifically, expert consensus guidelines recommend the use of multidisciplinary, individualized care as a first line approach to behavioral symptoms of dementia (Livingston *et al.*, 2005; O’Connor *et al.*, 2009; Burns *et al.*, 2012) which justified the inclusion of the two indicators relating to “alternatives” (i.e. trial of non-pharmacological therapy) and “indications” in our tool. Finally, there was strong consensus in the Delphi rounds that psychotropic use in people with dementia requires valid consent, if unable to be given by the person themselves, then by a proxy as determined hierarchically by Australian Guardianship laws (O’Neill and Peisah, 2011), a requirement often not adhered to in this setting (Gurian *et al.*, 1990; Rendina *et al.*, 2009).

In the setting of BPSD, we need to go beyond auditing with lists of medication, and use functional and interactive tools, not unlike the principle of the ARMOR tool (Assess – with Beers Criteria plus, Review, Minimize, Optimize, Reassess) which encourages a stepwise approach to polypharmacy in elderly persons using an interdisciplinary team-based approach (Haque, 2009). Haque (2009) found that after using the ARMOR in a geriatric rehabilitation and assessment setting there was a clear and consistent decline in the use of nine or more medications, and in the use of antipsychotics and antidepressants on their quality indicators. More specifically in regards to BPSD, the new DICE model (Describe, Investigate, Evaluate, and Create) seeks to reduce psychotropic medication use in people with dementia using an evidence-informed structured clinical reasoning process that can be integrated into diverse practice settings (Kales *et al.*, 2014).

The reduction in QUM- D scores suggests a change in prescriber behavior in a range of domains beyond drug choice, dose, consent, review, and use of non-drug alternatives, trialed in both inpatient and ambulatory settings, and most importantly, in the residential aged care facility setting. We also demonstrated its use by a range of disciplines, both nursing and medical. Feedback regarding its use suggested that skilled behavior management nursing staff who identified concerns regarding issues such as polypharmacy, could use the tool to empower their voice with prescribers. Indeed, notwithstanding the small numbers, the tool was used as an outcome measure/performance indicator by clinical nurse consultants for a BAMS. Additionally, it was used as a training tool for prescribing for registrars in old age psychiatry and with general practitioners.

We noted the resistance encountered in using the tool, mostly related to resource limitations within aged care facilities but also to poor understanding of family members and facility staff about the role and risks of psychotropics. This is consistent with previously identified barriers to the use of alternative approaches in managing BPSD including staff and resource limitations, lack of education and information about alternatives, environmental constraints, policy and management issues, beliefs, and expectations (of staff, family, and residents) (Moore and Haralambous, 2007). Specifically, lack of understanding about the potential side effects and consequences of use of psychotropics also contributes to their use (Agens, 2010). As illustrated in our case study, the tool has potential use in educating and empowering family members who put a lot of weight on the value of medications, while being ignorant of their potential harm and the value of non-pharmacological measures.

We concede a number of limitations to this tool. First, while we have attempted to validate its elements as representing a measure of quality prescribing by referencing the existing literature, we do not know if the tool does measure quality prescribing. Although the lack of comparable instruments limited the testing of concurrent validity of the whole instrument, component elements such as drug burden might have been validated by correlating QUM-D scores with adverse drug events. Despite the aim to be as inclusive and comprehensive as possible with generation of factors during the Delphi rounds, we may have overlooked important components of quality prescribing. For example, BPSD are often caused by underlying medical issues often missed when psychotropics are prescribed in a knee-jerk fashion. Secondly, in the absence of any patient data beyond diagnosis of dementia referred for treatment of BPSD, we do not know the efficacy of the tool for different severities or types of dementia. Having said this, the remit of the services upon which the tool was tested was to treat patients with severe BPSD. Thirdly, we had no data on performance of individual items. Fourthly, we had no control group to determine whether this reduction was associated with use of QUM-D *per se*. Fifthly, scores, although lowered, rarely returned to zero (the mean score at follow-up being 4.7, with one score post “intervention” at 16), suggesting a significant degree of resistance to de-prescribing, as described earlier. Sixthly, there is much subjectivity involved in the use of the “frail elderly specifier” and the perception of what constitutes excessive doses of antipsychotic drugs.

Finally, the QUM-D may cross-sectionally change prescribing behavior but we know little about long-term effects and a recent review suggested that empirical evidence regarding sustainability of interventions to reduce inappropriate prescribing of antipsychotic medications remains lacking (Thompson Coon *et al.*, 2014). Moreover, the Hawthorne effect may well have been operating such that prescribers, aware that they were being monitored for this trial, merely changed their behavior because, and while, they were being observed not because of the QUM-D *per se*. If the tool relied on some interactive component, as we demonstrated with use by nursing staff, this would be consistent with hypotheses that successful de-prescribing is reliant on an interactive and multi-disciplinary process, currently being tested in the recent de-prescribing studies (Smeets *et al.*, 2013; Brodaty *et al.*, 2014).

Despite these limitations, given the endemic nature of inappropriate prescribing and conceptualizing quality prescribing as a dimensional, not categorical concept, our goals were modest, such that if doses were reviewed for the first time and decreased slightly, or if family members were notified, or if a trial of person-centered care was attempted, or if simply the QUM-D caused a prescriber to put their mind to the question of drug use, we suggest that it is useful. The QUM-D may facilitate action in regards to prescribing in a quality direction (scores never increased). Arguably, fine tuning of what doses are acceptable or not, is not as important as triggering review and thought regarding the use of medication in this setting, particularly as people are often started on psychotropics and left on them indefinitely, or for long periods without review (Barnes *et al.*, 2012).

Useful future work would include correlations of scores with positive outcomes such as prescriber practices and knowledge transfer, and with negative patient outcomes such as hospital admission, mortality, and adverse events, which have been typically absent in all interventions to optimize prescribing for older people in care homes (Alldred *et al.*, 2013), as well as further exploration of resistance to de-prescribing.

The QUM-D is a tool which may help to improve the quality of prescribing practices of psychotropics in the context of BPSD. In this setting, we consider quality prescribing to be an inclusive concept beyond drug choice, beyond drug dose, beyond de-prescribing, beyond review, beyond ensuring that non-pharmacological options have been trialed, and beyond obtaining consent. We suggest that quality prescribing is all of these, as are the obligations of clinicians to patients with BPSD.

## Conflicts of interest

Carmelle Peisah and Henry Brodaty have received past research support and speaker's honoraria variously from Elis Lily, Astra Zeneca, Lundbeck, Novartis, Eisai, Janssen, and Pfizer.

## Description of authors’ roles

C. Peisah designed the study, supervised the data collection and wrote the paper. J. Strukovski supervised the data collection and wrote the paper. C. Wijeratne collected data and participated in the Delphi rounds and wrote the paper. R. Mulholland collected data and wrote the paper. G. Luscombe was responsible for the statistical design of the study and for carrying out the statistical analysis and wrote the paper. H. Brodaty participated in the Delphi rounds and wrote the paper.

## Appendix A: The quality use of medications in dementia (QUM-D) tool

**Table:**

***Quality parameter***	***Descriptor***	***Score***
**1. Alternatives**	No evidence of non-pharm trial	2
**2. Indication**	Use of drug without clear target symptom or for symptoms not considered targets for psychotropic use by consensual opinion e.g. wandering, screaming	2
**3. Choice of drug**	Use of drug with unsupported evidence base or lacking general consensus in this context e.g. sodium valproate, chlorpromazine, trifluoperazine, phenobarbitone.	2
	Use of drug otherwise contraindicated e.g. medication with high anticholinergic load in patient with retention/prostatism, use of medication with high insulin resistance in someone with diabetes, use of typical antipsychotics in Lewy body dementia	2
**4. Consent**	No evidence of consent documented from either person themselves or proxy as per hierarchy of NSW Guardianship Act 1987 (i.e. Guardian, Persons Responsible etc.). Include use of hormone treatment or opioids to treat behavior without documentation of consent.	2
**5. Dosage**	Use of doses* in excess of best practice guidelines e.g. Haloperidol > 2 mg * Olanzapine > 10mg * Risperidone > 2mg * Quetiapine > 200mg *	2
	Use of doses FAR in excess of best practice guidelines (NB score “2” in addition to scoring above item) e.g. Haloperidol ≥ 5mg daily Olanzapine ≥ 20 mg daily Risperidone ≥ 4 mg daily Quetiapine ≥ 300 mg daily	2
**6. Mode of administration**	Depot antipsychotic use	2
**7. Titration**	Rapid (e.g. usually no faster than every 3 days) – unless emergency	2
	Unreviewed – review must be for side effects (frequently e.g. every 3 days) and efficacy (after a week, or two/three for antidepressants) with dose adjustment	2
**8. Polypharmacy**	Use > 2 psychotropics simultaneously, including: prn medications if given, sedative/hypnotics such as temazepam, nitrazepam or flunitrazepam, oxazepam, clonazepam, and opioids	2
	Use > 4 psychotropics (NB score “2” in addition to scoring above item)	2
	Use of > 2 psychotropics for same drug class e.g. x 2 antipsychotics (NB score “2” in addition to scoring above item)	2
**9. Toxicity**	Evidence of side effects at a persistent or unacceptable level without review e.g. drowsiness, falls, EPSE, swallowing difficulties, QT prolongation	2
**10. Review**	Unreviewed continuous use of same drug > 3mths (review must constitute medical review including symptom review +/– trial of discontinuation) or continuous use of drug despite specialist advice or recommendation	2

Key: * = **Frail elderly specifier**. In frail elderly, with several co-morbid medical conditions, lower cap doses may be required and lower doses may be considered “burdensome.” For these patients, consider rating “2” for: haloperidol > 1mg; risperidone >1 mg, olanzapine >5 mg, quetiapine > 50 mg.

EPSE = extrapyramidal side effects.

## Appendix B: How to use the QUM-D

1. Question: What is it, and who is it for?
  Answer: It is a decision support aid for prescribers treating patients with dementia and BPSD who are currently on psychotropics.
  The aim is to reduce the score and in doing so to educate prescribers about medications often used in dementia, preferably as an adjunct to providing non-pharmacological treatments.
2. Question: How to use it?
  Answer: **score categorically (either 2 or 0) at first contact** with the patient or prior to any medication change. Repeat after you have intervened/reviewed/changed medications/documented consent etc.
  Exclude acetyl cholinesterase inhibitors
